# How Basic Can You Get?

**Published:** 1991-12

**Authors:** Richard Iles


### "HOW BASIC CAN YOU GET?"

Some 35 years ago I was on demob. leave from the RAMC, after three years in Kenya, and was awaiting a return passage to Kenya to take up a civilian post.

At short notice, I was asked to fill a week's gap in Locum cover for an elderly, single-handed, batchelor G.P. with a reputation for tight-fistedness. He had fallen down an 'economically' unlit flight of stairs at his house, where he lived 'over the shop', and was in hospital with a fractured neck of femur.

I visited Dr. X in hospital, where I was cautioned not to examine the patients too thoroughly "as they weren't used to it", and he added that if they had to undress he would be expected to provide extra heating in the consulting room. This, with a bare-boarded, stark waiting room, ("Last patient in please turn off the gas fire"), contrasted strongly with his residential quarters. I was further enjoined that "Any shillings for Private Certificates were **his** dues, not mine", and should be placed in a certain drawer in his desk.

Dr. X had resigned in protest at the inception of the N.H.S., and had taken early retirement, reputedly having made a ceremonial bonfire of all his books and papers. After a short while he had regretted this act and had returned to work, under the N.H.S. By then, only a few of his ex-patients returned to him, and his list was very small, certainly numbered in 3 figures only. As a consequence, his surgeries seldom exceeded 3 patients per session and on at least one occasion nobody came in at all; so I was hardly over-worked.

The Practice was in a densely-populated, inner-city district, within less than a mile of the main City Hospital, so his patients had been in the habit of taking themselves, direct to the Hospital's Casualty Dept. 'so as not to bother the Doctor', and emergency calls to Dr. X were few and far between.

Being between jobs, most of my medical equipment and any emergency drugs were in store, in Kenya, so I asked Dr. X if I could have the use of his emergency/night visit bag, should the necessity arise. He told me that his bag was on a chair in his dining room and that I should ask his housekeeper for it. I was assured that it contained "all that would be needed".

His housekeeper duly led me into his dining room where on one of the chairs was indeed a cheap, cardboard suit case with a red cross painted on the lid. After the bleak surgery premises I was surprised by the sumptuous furnishings and furniture: a beautiful mahogany table and sideboards, with a suite of six matching Chippendale chairs with two carvers, elegant silverware and expensive Wilton carpets complemented the opulence.

My second surprise was how light the case was. But surprise gave way to astonishment and incredulity when I opened the case.

I have already suggested that the contents represented the ultimate in 'Basic Medicine', and related the patients' habit of self-referral to the local Hospital, in emergency.

Dr. X was right, of course; in the special and peculiar context and circumstances of his practice, they **were** "all that was needed".

1. A STETHOSCOPE
2. A Book of Death Certificates.

Dr. Richard Iles

